# Reviewer acknowledgement 2013

**DOI:** 10.1186/1471-2164-15-205

**Published:** 2014-03-26

**Authors:** Irene Pala

**Affiliations:** 1 BioMed Central, 236 Gray’s Inn, Road, London WC1X 8HB, UK

## Abstract

**Contributing reviewers:**

The editors of BMC Genomics would like to thank all our reviewers who have contributed their time to the journal in Volume 14 (2013).

## 

Michael Abberton

Nigeria

Kamel Abd-Elsalam

Egypt

Ted Abel

USA

Mona Abirached-Darmency

France

Wolf-Rainer Abraham

Germany

Brett Abrahams

USA

Joel Acker

France

Herve Acloque

France

Heather Adams

USA

Charles Addo-Quaye

USA

Teresa Adell

Spain

Adebowale Adeyemo

USA

Nadav Ahituv

USA

Niyaz Ahmed

India

Dag Ahren

Sweden

Tahar Ait-Ali

UK

Nobuyoshi Akimitsu

Japan

Hasan Akman

USA

Sertac Aksakalli

Turkey

Fiammetta Alagna

Italy

Cedric Alaux

France

Peyman Alavi

Austria

M Mar Alba

Spain

Rob Alba

USA

Frank Albert

USA

Stefan Albert

Germany

Warren Albertin

France

Ute Albrecht

USA

Peter Alestrom

Norway

Andrey Alexeyenko

Sweden

Oleg Alexeyev

Sweden

Sher Ali

India

Pietro Alifano

Italy

Can Alkan

Turkey

Bassem Allam

USA

David Allred

USA

Ibrahim S Almssallem

Saudi Arabia

Enriqueta Alos

Spain

Adrian Altenhoff

Switzerland

Carlos Alvarez

USA

Sergio Alvarez

Chile

Lisa Alvarez-Cohen

USA

Nelson Alves Junior

Brazil

Karen Alviar

Philippines

Marcel Amills

Spain

Keenan Amundsen

USA

Gynheung An

Korea South

Cheryl Andam

USA

Alison Anderson

Australia

Leif Andersson

Sweden

Rune Andreassen

Norway

Jose Andres

Canada

Claudia Angelini

Italy

Franca Anglani

Italy

Ravindran Ankathil

Malaysia

Donna Ankerst

Germany

Matti Annala

Finland

Laima Antanaviciute

UK

Aldwin Anterola

USA

Christophe Antoniewski

France

Felipe Aquea

Chile

Manuel Aranda Lastra

Saudi Arabia

Marcos J. Arauzo-Bravo

Germany

Filippos Aravanopoulos

Greece

Tzahi Arazi

Israel

Michelle Arbeitman

USA

Hamutal Arbel

USA

Bruno Arcà

Italy

Patricio Arce-Johnson

Chile

David Archer

UK

Alan L. Archibald

UK

Paul Arens

Netherlands

Juan Lucas Argueso

USA

Peter Armbruster

USA

John Armour

UK

Ian Armstead

UK

Martha Arnaud

USA

Alain Arneodo

France

Catherine Arnold

UK

Ricardo Aroca

Spain

Katherine Aronstein

USA

Isabella Artner

Sweden

María Del Carmen Arufe Gonda

Spain

Kazuhide Asakawa

Japan

Ryan Ashley

USA

Herbert Auer

Spain

Raymond Auerbach

USA

Scott Auerbach

USA

Donald Auger

USA

Simon Avery

UK

Michael Axtell

USA

Belay Ayele

Canada

Julien Ayroles

USA

Rajeev Azad

USA

Vasco Azevedo

Brazil

David Azorsa

USA

Tomas Babak

USA

Vladimir Babenko

Russian Federation

Igor Babiak

Norway

Raman Babu

India

Francois Bachand

Canada

Maria Badenes

Spain

Katja Baerenfaller

Switzerland

Ewout Baerveldt

Netherlands

Vesselin Baev

Bulgaria

Angshuman Bagchi

India

Alessandro Bagnato

Italy

Hua Bai

USA

Matthew Bainbridge

USA

Harsh Bais

Sudan

Lauren Bakaletz

USA

Michael Baker

USA

Michelle Baker

Australia

Alma Balestrazzi

Italy

David Baltrus

USA

Stelvio Bandiera

Canada

Mukesh Bansal

USA

Leandra Baptista

Brazil

Pankaj Barah

Norway

Simon Barak

Israel

Abdelali Barakat

USA

Pavel Baranov

Ireland

Matthew Baranski

Norway

Yoseph Barash

USA

Daniel Barbash

USA

William Barbazuk

USA

Nuno L Barbosa-Morais

Portugal

William Barendse

Australia

Luca Bargelloni

Italy

Daniel Barker

UK

Guy Barker

UK

Keith Barker

USA

Jeffrey Baron

USA

Francisco Barona-Gomez

Mexico

Rodolphe Barrangou

USA

Hugo A Barrera-Saldaña

Mexico

Felipe Barreto

USA

Francisco Barro

Spain

Gerard Barroso

France

Cornelius Barry

USA

Brenda Bass

USA

Christoph Basse

Germany

Roberto Bassi

Italy

Ugo Bastolla

Spain

Arsen Batagov

Singapore

Patrick Bateson

UK

Rita Batista

Portugal

Jacqueline Batley

Australia

Jyotsna Batra

Australia

Cristina Battaglia

Italy

Natalia Battchikova

Finland

John Battista

USA

Peggy Baudouin-Cornu

France

Denis Carolin Jessica Bauer

Australia

Iliana Baums

USA

Miguel Beato

Spain

Ingeborg Becker

Mexico

Gary Beecham

USA

Shahriar Behboudi

UK

Marcel Behr

Canada

Susanta Behura

USA

Sebastian Beier

Germany

Stefan Bekiranov

USA

Jordana Bell

UK

Xavier Belles

Spain

Shayne Bellingham

Australia

Alexa Bely

USA

Choukri Ben Mamoun

USA

Abdelhafid Bendahmane

France

Asa Ben-Hur

USA

Chris Benner

USA

Christopher Benner

USA

Touati Benoukraf

Singapore

Jagadish Bentur

India

Caroline Berard

France

Gregory Berger

USA

Nicholas Bergman

USA

Tobias Bergmiller

Austria

Sara Bergonzi

Germany

Rodrigo Bermejo

Spain

Giorgio Bernardi

Italy

Maria Lina Bernardini

Italy

Claire Bertelli

Switzerland

Frederic Bertels

Switzerland

Thomas Bettecken

Germany

Carmen Beuzón

Spain

Andreas Beyer

Germany

Sukesh Bhaumik

USA

Vera Bianchi

Italy

Luca Bianco

Italy

Derek Bickhart

USA

Christopher Bidwell

USA

Gerd Patrick Bienert

Germany

Kyle Biggar

Canada

Patrick Biggs

New Zealand

Fabiana Bigi

Argentina

Jeannette Bigler

USA

Andras Bilkei-Gorzo

Germany

Emanuele Biondi

France

Inanc Birol

Canada

Danny Asher Bitton

UK

Michael Black

Australia

Lionel Blanchet

Netherlands

Mark Blaxter

UK

Marc Blondel

France

Marc Blondel

France

Burt Bluhm

USA

Yaroslav Blume

Ukraine

Miroslav Blumenberg

USA

Julien Bobe

France

Jon Bohlin

Norway

Joerg Bohlmann

Canada

Didier Boichard

France

Stephane Boissinot

USA

Matei Bolborea

UK

Hanna Bolibok-Bragoszewska

Poland

Alexander Bolshoy

Israel

Sebastian Boltana

UK

Ian Bonapace

Italy

Claudio Bonghi

Italy

Mariangela Bonizzoni

USA

Agnes Bonnet

France

Anne Bonnieu

France

Lori Bonnycastle

USA

Hidemasa Bono

Japan

James Bono

USA

Jeffrey Boore

USA

Stephanie Booth

Canada

Mark Borodovsky

USA

Sibele Borsuk

Brazil

Kirill Borziak

USA

Steven Bosinger

USA

Paul Boss

Australia

Mirte Bosse

Netherlands

Jose Botella

Australia

Anna Maria Botha Oberholster

South Africa

Arnaud Bottin

France

Nicolas Bouche

France

Pierre Boudinot

France

Pierre Boudry

France

Elizabeth Boulding

Canada

Tristan Boureau

France

Freddy Boutrot

UK

Marc Boutry

Belgium

Arnaud Bovy

Netherlands

Megan Bowman

USA

Kiymet Bozaoglu

Australia

Zbynek Bozdech

Singapore

Holger Brüggemann

Denmark

Cameron Bracken

Australia

Stephen Bradford

Australia

Dan Bradley

Ireland

Rosie Bradshaw

New Zealand

William Bradshaw

USA

Katharina Braeutigam

Canada

Rogelio Brandao

Brazil

Jose Bras

UK

Edward Braun

USA

Rudiger Brauning

New Zealand

Claudio Bravi

Argentina

Kelly Brayton

USA

Bronislava Brejova

Slovakia

Ghislain Breton

Canada

Ghislain Breton

USA

Holger Breuninger

UK

Diego Breviario

Italy

Susan Bridges

USA

Scott Briggs

USA

Jennifer Brisson

USA

Collette Britton

UK

Marcelo Brocchi

Brazil

Luciano Brocchieri

USA

Gudrun Brockmann

Germany

Gordon Broderick

Canada

Giovanni Broggini

Switzerland

Roland Brosch

France

James Brown

USA

Susan Brown

USA

William Browne

USA

Can Bruce

USA

Thiago Bruce

Brazil

Søren Brunak

Denmark

Joseph Brunelli

USA

Frederic Brunet

France

Vincent Bruno

USA

Josephine Bryant

UK

Ola Brynildsrud

Norway

Rong Bu

Saudi Arabia

Vicky Buchanan-Wollaston

UK

Carmen Buchrieser

France

Brad Buckley

USA

Hikmet Budak

Turkey

C. Robin Buell

USA

Alexander Bukreyev

USA

Andreas Buness

Germany

Francesco Buonocore

Italy

Gertraud Burger

Canada

Robert Burne

USA

Kelley Burridge

USA

Pierre Bushel

USA

Kathryn Bushley

USA

Matteo Buti

Italy

Geraldine Butler

Ireland

Michael Butterfield

Brazil

Thomas Butts

UK

Ramesh Buyyarapu

USA

Gregory Buzard

USA

Nicole Bye

Australia

Thomas Byrd

USA

Leonid Bystrykh

Netherlands

Mark Caddick

UK

Jean Lud Cadet

USA

Raquel Cadete

Brazil

Shibin Cai

China

Wanzhi Cai

China

Weili Cai

USA

Yudong Cai

China

Elisabet Caler

USA

Ann Callahan

USA

Juan Calvete

Spain

Stefano Calza

Italy

Jennifer Cameron

USA

Tolga Can

Turkey

Francisco R Canton

Spain

Tobias Cantz

Germany

Bihao Cao

China

Silvia Cardona

Canada

Robert Carey

USA

Jason Carlyon

USA

Margaret Carrel

USA

Laura Carreto

Portugal

Jason Carroll

UK

David Carter

UK

Kim Warwick Carter

Australia

Oliviero Carugo

Austria

Michael Carvan

USA

Sergi Castellano

Germany

Robert Castelo

Spain

Yves Castonguay

Canada

Luigi Cattivelli

Italy

Mathilde Causse

France

Pablo Federico Cavagnaro

Argentina

Joan Cerda

Spain

Luigi Cerulo

Italy

Alessandro Cestaro

Italy

Pieter-Jan Ceyssens

Belgium

Marie Chabbert

France

Subhra Chakraborty

India

Daniel Chamovitz

Israel

Frances Champagne

USA

Agnes Chan

USA

Pritam Chanda

USA

Jeff Chang

USA

Mark Chapman

UK

Jean-Philippe Charles

France

Brian Charlesworth

UK

Keith Chater

UK

Debasis Chattopadhyay

India

Harsh Chauhan

Switzerland

Sebastian Chavez

Spain

Anshu Chen

USA

Congying Chen

China

Fadi Chen

China

Feng Chen

USA

Hao-Tai Chen

China

Jin Chen

USA

Ming Chen

China

Mingsheng Chen

China

Ming-Shun Chen

USA

Rongjun Chen

China

Shilin Chen

China

Ting Chen

USA

Wei Chen

Germany

Wei Chen

China

Weiping Chen

USA

Xi Chen

China

Yo-Chia Chen

Taiwan

Chao Cheng

USA

Chun-Wen Cheng

Taiwan

Feng Cheng

China

Hans Cheng

USA

Zhukuan Cheng

China

Zachary Cheviron

USA

Alan Chiang

China

Yu-Chung Chiang

Taiwan

Yukako Chiba

Japan

Koen Chiers

Belgium

Chi-Chou Chiu

Taiwan

Michael Cho

USA

Doil Choi

Korea South

Hong-Kyu Choi

Korea South

Kang Chong

China

Valerie Choumet

France

Brock Christensen

USA

Yeun-Jun Chung

Korea South

Gary Churchill

USA

Angelica Cibrian

Mexico

Alfredo Ciccodicola

Italy

Daniel Ciobanu

USA

Chiara Cirelli

USA

Daniela M Cirillo

Italy

Melody Susan Clark

UK

Taane Clark

UK

Christopher Clarke

USA

Rainer Claus

Germany

Thomas Clavel

Germany

David Clayton

UK

Maysa Clementino

Brazil

Beth Cleveland

USA

Timothy Close

USA

Peter Clote

USA

Steve Clough

USA

Gitta Coaker

USA

Geoffrey Coast

UK

Benjamin Cocks

Australia

Sergio Cocozza

Italy

Susana Coelho

France

Patricia Coello

Mexico

Thom Colgan

Ireland

Anne Collin

France

Alison Collins

Australia

Amedeo Columbano

Italy

David Comas

Spain

Inaki Comas

Spain

Tim Connallon

USA

Erin Connor

USA

Greg Cook

New Zealand

Jonna Coombs

USA

Vaughn Cooper

USA

Mark Corbett

Australia

Jonathan Corcoran

UK

Cynthia Cornelissen

USA

Antonio Correia

Portugal

Alfred Cortes

Spain

Fabrizio Costa

Italy

Antonio Costa De Oliveira

Brazil

Lucía Couceiro

France

Christine Couldrey

New Zealand

Brian Counterman

USA

B. Cournoyer

France

Juan Pablo Couso

UK

Patrick Covello

Canada

Russell Cox

Albania

David Craig

USA

Robert Cramer

USA

Jacob Crawford

USA

Helen Crooke

UK

Lisa Crossman

UK

Sean Crosson

USA

Nicholas Croucher

USA

Qinghua Cui

China

Dan Cullen

USA

Andrew Charles Cuming

UK

Anderson Cunha

Brazil

Luis Cunha

Brazil

Sébastien Cunnac

France

Francis Cunningham

USA

Edwin Cuppen

Netherlands

Thomas Curie

Switzerland

Cameron Currie

USA

Graziella Curtale

Italy

Salvatore Cuzzocrea

Italy

Artur Da Câmara Machado

Portugal

Jesse Dabney

Germany

Nunzio D'Agostino

Italy

Silan Dai

China

Yang Dai

USA

Lisa Dailey

USA

Emmanuelle D'Alençon

France

Yuri D'Alessandra

Italy

Brian Dalrymple

Australia

Thomas Dandekar

Germany

Phat Dang

USA

Roy Danzmann

Canada

Sujoy Das Gupta

India

Mark D'Ascenzo

USA

Raju Datla

Canada

Brigitte Dauwalder

USA

Mark Davey

Belgium

William Davidson

Canada

Mark Davies

UK

Gregory Davis

USA

Thomas Davis

USA

Roberta Davoli

Italy

Paul De Bakker

USA

Sophie De Bentzmann

France

Michele De Bortoli

Italy

Marc De Braekeleer

France

Robert De Bruin

UK

Carolyn De Graaf

Netherlands

Dirk De Graaf

Belgium

Anne De Jong

Netherlands

Monique De Jong

Netherlands

Ronnie De Jonge

Netherlands

Annelies De Klein

Netherlands

Alexandre De Kochko

France

Dirk-Jan De Koning

Sweden

Xavier De La Cruz

Spain

Wanessa De Lima

Switzerland

Ruud De Maagd

Netherlands

Joao Pedro De Magalhaes

UK

Louisi De Oliveira

Brazil

Emanuele De Paoli

Italy

Valli De Re

Italy

Robson Francisco De Souza

Brazil

Ronald De Vries

Netherlands

Jeremy Debarry

USA

Thomas Debener

Germany

Franck Dedeine

France

Patrick Degnan

USA

Sandie Degnan

Australia

Rosa Del Campo

Spain

Clelia De-La-Pena

Mexico

Nicholas Delihas

USA

Silvia Della Bella

Italy

Christine Delorme

France

Regine Delourme

France

Constantinos Deltas

Cyprus

Laurent Deluc

USA

Hendrik Cornelis Den Bakker

USA

Chuxia Deng

USA

Hong-Wen Deng

USA

Paul Denny

UK

Thomas Derrien

France

Rob Desalle

USA

Michael Desalvo

USA

Patrick Descombes

Switzerland

Christopher Desjardins

USA

Christine Desmedt

Belgium

Yves Dessaux

France

H. William Detrich

USA

Adam Deutschbauer

USA

Yvan Devaux

Luxembourg

Colin Dewey

USA

Frederick Dewey

USA

Michael K Deyholos

Canada

Zoltan Dezso

USA

Swatismita Dhar

India

Angelique D'Hont

France

Paola Di Meglio

UK

Pier Paolo Di Nocera

Italy

Antonio Di Pietro

Spain

Iulia Diaconu

USA

Fernando Diaz

Spain

Oscar Diaz Horta

USA

Thomas Dick

Singapore

Giorgio Dieci

Italy

Sarah Diermeier

USA

Christoph Dieterich

Germany

Rod Dimaline

UK

Emmanuel Dimont

USA

Scott Dindot

USA

Hu Ding

China

Lianghao Ding

USA

Nengshui Ding

China

Yi Ding

China

Ottmar Distl

Germany

Oliver Distler

Switzerland

Dirk Dittmer

USA

Brian Dixon

Canada

Hakim Djaballah

USA

Sarah Djebali

Spain

Alexander Dobin

USA

Harshavardhan Doddapaneni

USA

Ken Dodds

New Zealand

Jerry Dodgson

USA

Andrew Doig

UK

Jaroslav Dolezel

Czech Republic

Xavier Donadeu

UK

Claudio Donati

Italy

Chun-Hai Dong

China

Nilgun Donmez

Canada

Martin Donnelly

UK

Joaquin Dopazo

Spain

Charles J. Dorman

Ireland

Anne Dorrance

USA

Patricia Dos Santos

USA

Scott Dougan

USA

Carl Douglas

Canada

Robin Dowell

USA

Tim Downing

Ireland

Tommaso Dragani

Italy

Jennifer Drake

USA

Michel Drancourt

France

Damian Drew

Australia

Tom Druet

Belgium

Delin Duan

Cocos (Keeling) Islands

William Duax

USA

Benjamin Dubansky

USA

Inna Dubchak

USA

Geraldine Dubreuil

France

Andrew Dudley

USA

Edward Dudley

USA

Joel Dudley

USA

Patrick Duffy

USA

Sandra Duharcourt

France

Alexandra Dumitriu

USA

Keith Dunker

USA

Christopher Dunlap

USA

Casey Dunn

USA

Ian Dunn

UK

Paul Dunn

New Zealand

Susana Dunner

Spain

Pascal Durrens

France

Bas E. Dutilh

Netherlands

Chitra Dutta

India

Michael Dyck

Canada

Paul Dyson

UK

Tai E Shyong

UK

Andrew Eamens

Australia

Ingo Ebersberger

Germany

Jeanette Eckel-Passow

USA

Konstantinos Economopoulos

USA

Eric Edsinger

Japan

David Edwards

Australia

Owain Edwards

Australia

Shigeki Ehira

Japan

Armin Ehrenreich

Germany

Garth Ehrlich

USA

Juergen Eils

Germany

Eli Eisenberg

Israel

Marc Ekker

Canada

Michael Elashoff

USA

Michelle Elekonich

USA

Simon Ellwood

Australia

Osman El-Maarri

Germany

Paula Elomaa

Finland

Christine Elsik

USA

Benjamin Elsworth

UK

Francesco Emanuelli

Italy

Richard Emes

UK

Scott Emrich

USA

Yuichi Endo

Japan

Luis Enjuanes

Spain

Jacob Enk

Canada

Mark Eppinger

USA

Sevinc Ercan

USA

Maria Raffaella Ercolano

Italy

Henry Erlich

USA

Yaniv Erlich

USA

Uli Ernst

Belgium

Lennart Eschen-Lippold

Germany

Matthew Escobar

USA

Jorge Esparza Gordillo

Germany

Luca Espen

Italy

Yolanda Espinosa Parrilla

Spain

M Angeles Esteban

Sudan

Anna Esteve

Spain

Jason Evenhuis

USA

Eduardo Eyras

Spain

Ines Ezcurra

Sweden

Hiroshi Ezura

Japan

João Fadista

Denmark

Noah Fahlgren

USA

Birthe Fahrenkrog

Belgium

Germana Falcone

Italy

Jian-Bing Fan

USA

Longjiang Fan

China

David Fang

USA

Gang Fang

USA

Cathy Sj Fann

Taiwan

Kerrie Farrar

UK

Paul Farrell

UK

Marianna Fasoli

Italy

Mathias Faure

France

Herbert Federhen

USA

Natalie Fedorova Abrams

USA

Christine Fehlner-Gardiner

Canada

Mark Feinberg

USA

Kyriacos Felekkis

Cyprus

Kurt Fellenberg

Germany

F. Alex Feltus

USA

Liane Fendt

Austria

Qili Feng

China

Anne Fennell

USA

Francesco Feo

Italy

Jorge Fernandes

Norway

Olivier Feron

Belgium

Serena Ferraresso

Italy

Alberto Ferrarini

Italy

Henrique Ferreira

Brazil

David Ferrier

UK

Howard W. Fescemyer

USA

Jennifer Fettweis

USA

Mark Field

UK

Agnes Figueiredo

Brazil

Melanie Filiatrault

USA

Guillaume Filion

Spain

Alain Filloux

UK

Antje Fischer-Rosinský

Germany

Elisabeth Fitzek

USA

J Ross Fitzgerald

UK

Lex Flagel

USA

Klas Flardh

Sweden

Joost Fledderus

Netherlands

Michelle Flenniken

USA

Delphine Fleury

Australia

Paul Flicek

UK

Laurence Flori

France

Marco Fondi

Italy

Claudia Fonseca

Brazil

Marbella Fonseca

Brazil

Paolo Fontana

Italy

Luca Fontanesi

Italy

Christiane Forestier

France

Sylvain Forêt

Australia

John Forster

Australia

Marina Fortes

Australia

Louis-Charles Fortier

Canada

Nicholas Foulkes

Germany

Howard Fox

USA

Jay Fox

USA

Olga Francino

Spain

Christof Francke

Netherlands

Octavio Franco

Brazil

Jose Franco Da Silveira

Brazil

Lionel Frangeul

France

Philipp Franken

Germany

Adam Frankish

UK

Paul Fraser

UK

Ducancel Frédéric

France

Reid Frederick

USA

Erik Fredlund

Sweden

David Fredman

Austria

Maria Fredriksson-Ahomaa

Finland

Jonathan Freedman

USA

Lloyd Fricker

USA

Marc Friedlander

Spain

Stephanie Friedman

USA

Thomas Friedman

USA

Gerhard Fritz

Germany

Rebecca Fry

USA

William Fry

USA

Jinmin Fu

China

Hiroaki Fujii

Finland

Toshinobu Fujiwara

Japan

Tatsuo Fukagawa

Japan

Kenji Fukunaga

Japan

Yoshifumi Fukunishi

Japan

Atsushi Fukushima

Japan

Melissa Fullwood

Singapore

Vilmos Fulop

UK

Eileen Furlong

Germany

Naoyuki Fuse

Japan

Idan Gabdank

USA

Macoura Gadji

Canada

Gabor Galiba

Hungary

Marie-Dominique Galibert

France

Leonardo Galindo

Canada

Eric Gamazon

USA

Anand Ganesan

USA

Eric Ganko

USA

Victor Gannon

Canada

Wenxiang Gao

China

Zhihong Gao

China

Nigel Gapper

USA

Gizele Garcia

Brazil

Juan Antonio Garcia

Austria

Elsa Garcia-Gamez

Spain

Jordi Garcia-Mas

Spain

Donald Gardiner

Australia

Katheleen Gardiner

USA

Susan Gardiner

New Zealand

Chad Garner

USA

Manuel Garrido-Ramos

Spain

Christine Gaspin

France

Francisco Gasulla

Spain

Kalamegam Gauthaman

Saudi Arabia

Mathieu Gautier

France

Philippe Gayral

France

Isabella Gazzoli

Netherlands

Yvette Gbelska

Slovakia

Xijin Ge

USA

Rodney Geisert

USA

Mikhail Gelfand

Russian Federation

Eileen Gentleman

UK

Emmanuel Geoffriau

France

Christos Georgiou

Greece

Dan Geraghty

USA

Anna Gerasimova

USA

Daniel Gerlach

Austria

Shanaz Ghandhi

USA

Gargi Ghosal

USA

Debashis Ghosh

USA

Zhumur Ghosh

India

Sergio Giannattasio

Italy

Gillian Gibb

New Zealand

John Gibbons

USA

Lisle Gibbs

USA

Patrick Gibney

USA

Miriam Gifford

UK

Mark Gijzen

Canada

Bikram Gill

USA

Steven Gill

USA

David Gillan

Belgium

Michael Gilmore

USA

Aurélien Ginolhac

Denmark

Ricardo Giordano

Brazil

Jim Giovannoni

USA

Massimo Giovannotti

Italy

Santhosh Girirajan

USA

Carmela Gissi

Italy

Matt Gitzendanner

USA

Liz Glass

UK

Frank Oliver Glockner

Germany

Gernot Gloeckner

Germany

Kevin Alan Glover

Norway

Yasuhiro Go

Japan

Philip Goetz

USA

Omer Gokcumen

USA

James Golden

USA

Yury Goltsev

USA

Katsuya Gomi

Japan

Michael Gonda

USA

Florence Gondret

France

Cedric Gondro

Australia

Chengliang Gong

China

Elsa Gongora

USA

Carlos Gonzalez

USA

Zinnia Gonzalez Carranza

UK

Jose L. Gonzalez Hernandez

USA

David Goode

Australia

Michael Goodfellow

UK

Michele Goodhardt

France

Dmitry Gordenin

USA

David Gordon

Australia

Karl Gordon

Australia

Kevin Gori

UK

Ivan Gorlov

USA

Johanna Gostner

Austria

Manfred Grabherr

Sweden

Kirsi Granberg

Finland

Atle Van Beelen Granlund

Norway

Barbara Gravendeel

Netherlands

Christian Gray

UK

Sol Green

New Zealand

Catherine Greene

Ireland

Gustavo Bueno Gregoracci

Brazil

Brian Gregory

USA

T. Ryan Gregory

Canada

Ruth Grene

USA

Laura Grenville-Briggs

Sweden

Peter Gresshoff

Australia

Darren Griffin

UK

Obi Lee Griffith

USA

Igor Grigoriev

USA

Raymond Grill

USA

Jerome Grimplet

Spain

Nigel Harry Grimsley

France

Paul E. Grini

Norway

Edmundo Grisard

Brazil

Martien Groenen

Netherlands

Astrid T. Groot

Germany

Marco Groth

Germany

Anne Grove

USA

Anil Grover

India

Christina Grozinger

USA

Kristina Gruden

Slovenia

Rebecca Grumet

USA

Leluo Guan

Canada

Rafael Guedes

Brazil

Alfredo Guéra

Spain

Muriel Gugger

France

Roderic Guigo

UK

Yann Guiguen

France

Tom Guilfoyle

USA

Patricia M Guimaraes

Brazil

Per Guldberg

Denmark

Maria Gullo

Italy

An-Yuan Guo

China

Da-Long Guo

China

Zhigang Guo

USA

Hari Gupta

India

Priyatansh Gurha

USA

Victor Guryev

Netherlands

Alfonso Gutierrez-Adan

Spain

Jose Gutierrez-Marcos

UK

Armel Guyonvarch

France

Carlos Guzman

Spain

Christoph Haag

Switzerland

Eric Haag

USA

Brian Haas

USA

Tsuyoshi Hachiya

Japan

Michael Hackenberg

Spain

Perry Hackett

Antarctica

Hubert Hackl

Austria

Steve Haddock

USA

Houria Hadj-Arab

Algeria

Stephan Hahn

Germany

Candace Haigler

USA

Marielle Haks

Netherlands

Matthew Hale

USA

Chris Haley

UK

Michael Hall

Switzerland

Sarah Hall

USA

Pille Hallast

UK

Jorg Hamann

Netherlands

Stavros Hamodrakas

Greece

Miriam Hampel

Spain

Dong Wook Han

Korea South

Jiali Han

USA

Jianyong Han

China

Min Han

USA

Weiguo Han

USA

Yuepeng Han

China

Yujun Han

USA

Hirokazu Handa

Japan

Alfred Handler

USA

Oliver Hankinson

USA

Ebert Hanna

Brazil

Anthony Hannan

Australia

Sridhar Hannenhalli

USA

J Hansen

USA

Christian Hardtke

Switzerland

Barbara Harlizius

Netherlands

Alice Harmon

USA

Steven Harris

USA

Andrew Harrison

UK

Richard Harrison

UK

Edgar Hartsuiker

UK

Tadao Hasegawa

Japan

Karl Hassan

Australia

Martin Hasselblatt

Germany

Graham Hatfull

USA

Ricardo Hattori

Japan

Bettina Hause

Germany

Loren Hauser

USA

Jennifer Hawkins

USA

Cheryl Hayashi

USA

Ben Hayes

Australia

Patrick Hayes

USA

Alice Hayward

Australia

Chaozu He

China

Dan He

USA

Fuchu He

China

Guanghua He

China

Pingan He

China

Ping-An He

China

Xionglei He

China

Yuke He

China

Denis Headon

UK

Julia Heck

USA

Dennis Hedgecock

USA

Harald Hedman

Sweden

Charlotte Hedskog

USA

Dwayne Hegedus

Canada

Mohammad Heidari

USA

Matthias Heinig

Germany

Knut J. Heller

Germany

Katarzyna Heller-Uszynska

Australia

Chris Helliwell

Australia

Adriana Hemerly

Brazil

Richard Hemmi Valente

Brazil

John Hempel

USA

Robert Henry

Australia

Theodore Henry

UK

John Henshall

Australia

Yann Herault

France

Juan Hermoso

Spain

Pilar Hernandez

Spain

Michel Hernould

France

Amaury Herpin

Germany

Alfredo Herrera-Estrella

Mexico

Jean-Louis Herrmann

France

Paul Herron

UK

Klemens Hertel

USA

Ralf Herwig

Germany

Michel Herzog

France

Wolfgang Rene Hess

Germany

Martin Hetzer

USA

David Hibbett

USA

André Hidalgo

Netherlands

Atsunori Higashino

Japan

Dominique Higuet

France

Kenji Hikosaka

Japan

Karen Hill

USA

Heather Hines

USA

Junya Hiroi

Japan

Kiichi Hirota

Japan

Birte Höcker

Germany

Anne Hocking

USA

Jean-Francois Hocquette

France

Ludwig Eduard Hoelzle

Germany

Katharina Hoff

Germany

Michael Hoffman

Canada

Thomas Hoffmann

USA

Dirk Hoffmeister

Germany

Michael Hofreiter

UK

Matthew Holden

UK

Mitchell Holland

USA

Ed Hollox

UK

Mark Holmes

UK

Kathryn Holt

Australia

Gary Hon

USA

Saul Honigberg

USA

Derek William Hood

UK

Jan-Olov Höög

Sweden

Jayne Hope

UK

Andrew Hopkinson

UK

Tiago Hori

Canada

Toshihiro Horiguchi

Japan

Petr Horin

Czech Republic

Fumihiko Horio

Japan

David Peter Hornby

UK

Emily Hornett

USA

Eran Hornstein

Israel

Nelson Horseman

USA

David Horvath

USA

Benjamin A. Horwitz

Israel

Xin Hou

USA

Andreas Houben

Germany

Ross Houston

UK

Bjorn Hoyheim

Norway

Eva Hribova

Czech Republic

Chia-Lang Hsu

Taiwan

Juchun Hsu

Taiwan

Kui-Ching Hsu

Taiwan

Jinguo Hu

USA

Min Hu

China

Ming Hu

USA

Tinghui Hu

USA

Bangquan Huang

China

Bevan Emma Huang

Australia

Gang Huang

USA

Jin Huang

USA

Jingshan Huang

USA

Lusheng Huang

China

Shaobai Huang

Australia

Xi Huang

China

Xudong Huang

USA

Yongping Huang

China

Nicholas James Hudson

Australia

Isabelle Hue

France

Jim Hughes

UK

David Hume

UK

Christian Humpel

Austria

David Humphreys

Australia

Eric Hungate

USA

Katherine Hunt

USA

Martin Hunt

UK

Peter Hunt

Australia

Brandon Hurr

USA

Stephan Hutter

Germany

Gyorgy Hutvagner

Australia

Arnaud Huvet

France

Ui-Wook Hwang

Korea South

Doug Hyatt

USA

Michael F. Hynes

Canada

Naoko Imamoto

Japan

Richard Immink

Netherlands

Juan Imperial

Spain

Anastasia Imsiridou

Greece

Aaron Ingham

Australia

Massimo Iorizzo

USA

Manuel Irimia

Canada

Iker Irisarri

Spain

Iker Irisarri

Germany

Kazuyuki Ishihara

Japan

Masakazu Ishikawa

Japan

Yoshiaki Ito

Singapore

Takeshi Itoh

Japan

Yuka Iwasaki

Japan

Lakshminarayan Iyer

USA

Eva Jablonka

Israel

Rogerio Jacinto

Brazil

Michael Jackson

UK

Scott Jackson

USA

William Jacobs

USA

Guru Jagadeeswaran

USA

Mukesh Jain

India

Pankaj Jaiswal

USA

Euan James

UK

Hélène Jammes

France

Guilhem Janbon

France

Sarath Chandra Janga

USA

Michal Jank

Poland

Lukasz Jaskiewicz

Switzerland

Katy Jeannot

France

Zeenath Jehan

Saudi Arabia

Lars Jensen

Denmark

Tim K. Jensen

Denmark

Dong-Hoon Jeong

USA

Haeyoung Jeong

Korea South

Aaron Jex

Australia

Chen Jiajia

China

Cai-Zhong Jiang

USA

Lixi Jiang

China

Ning Jiang

USA

Rays Jiang

USA

Shuye Jiang

Singapore

Yiwei Jiang

USA

Yuanqing Jiang

China

Zhou Jian-Hua

China

Yuannian Jiao

USA

Guohua Jin

China

Xin Jin

China

Rolf Joerger

USA

Johann Joets

France

Urban Johanson

Sweden

Stewart Johnson

Canada

Timothy Johnson

USA

Elisabeth Jonas

Sweden

Alex Jones

UK

Brian Jones

Australia

Gary Jones

Ireland

Peter Jordan

Portugal

Hossein Jorjani

Sweden

Jingfang Ju

USA

Liran Juan

China

Howard Judelson

USA

Matthieu Jules

France

Ki-Hong Jung

Korea South

Sook Jung

USA

Haja Kadarmideen

Denmark

Koji Kadota

Japan

Yuji Kageyama

Japan

Minna Kaikkonen

Finland

Hironaga Kakoi

Japan

Thomas Källman

Sweden

Stanislaw Kaminski

Poland

Sophien Kamoun

UK

Yunchao Kan

China

Bavesh Davandra Kana

South Africa

Akio Kanai

Japan

Angelos Kanellis

Greece

Hyun Ah Kang

Korea South

Jiuhong Kang

China

Zhensheng Kang

China

Merijn Kant

Netherlands

Juha Kantanen

Finland

Levente Karaffa

Hungary

Emre Karakoc

USA

Emmanuel Kas

France

Khalil Kashkush

Israel

Michael Katze

USA

Ibrahim Halil Kavakli

Turkey

Mari Kawaguchi

Japan

Hideya Kawaji

Japan

Kanako Kawaura

Japan

Ehsan Kayal

USA

Xisong Ke

Norway

Orla Keane

Ireland

John William Keele

USA

Julia Kehr

Spain

Julia Kehr

Germany

Bart Keijser

Netherlands

Beat Keller

Switzerland

Joanna Kelley

USA

Laura Kelly

UK

Gerrit Haatje Jan Kema

Netherlands

Kathryn Kemper

Australia

Carly Kenkel

USA

John Kennell

USA

Jane Kenney-Hunt

USA

Nathan Kenny

UK

Paul Kersey

UK

Hindrik Harm Dirk Kerstens

Netherlands

James Ketudat Cairns

Thailand

Brian Kidd

USA

Alexandra K. Kiemer

Germany

James Wraith Kijas

Australia

Shoshi Kikuchi

Japan

Andrzej Kilian

Australia

Mogens Kilian

Denmark

Hyeran Kim

Korea South

Kwan Suk Kim

Korea South

Seongho Kim

USA

Seongho Kim

USA

Robin Kimmel

Austria

Kati Kinnunen

Finland

Martin Kircher

USA

Hisanori Kiryu

Japan

Uday Kishore

UK

Jun Kitano

Japan

Sabine Klauck

Germany

Mikhail Klenov

Russian Federation

Daniel Kliebenstein

USA

Karin Klinga-Levan

Sweden

Jens Klockgether

Germany

Robert Klopfleisch

Germany

Sandra Knapp

UK

Volker Knoop

Germany

Joshua Knowles

USA

Ichizo Kobayashi

Japan

Thomas Kocher

USA

Alex Kochetov

Russian Federation

Karin Koehl

Germany

Sunitha Kogenaru

USA

Michael Hans Kohn

USA

Narasaiah Kolliputi

USA

James Koltes

USA

Kostas Konstantinidis

USA

David Kopecky

Czech Republic

Jan Korbel

Germany

Ian Korf

USA

Nicholas Korir

Kenya

Cindy Körner

Germany

KlÁRa KosovÁ

Czech Republic

Sergei Kosushkin

Russian Federation

Masaaki Kotera

Japan

Martha Koukidou

UK

Piotr Kozlowski

Poland

Elena Kramer

USA

Frank Kramer

Germany

Aleksei Krasnov

Norway

Lukas Kratochvil

Czech Republic

Grigorios Krey

Greece

Carsten Kröger

Ireland

Andrew Kropinski

Canada

Marija Krstic-Demonacos

UK

Markus Krupp

Germany

Jaroslaw Krzywinski

UK

Michal Ksiazkiewicz

Poland

Christian P. Kubicek

Austria

Mikael Kubista

Sweden

Katashi Kubo

Japan

Karl Kuchler

Austria

Christa Kuehn

Germany

Larry Kuehn

USA

Ursula Kües

Germany

Jan Kuever

Germany

Heiner Kuhl

Germany

Leonid Kulakov

UK

Amar Kumar

India

Dinesh Kumar

India

Sudip Kundu

India

Tanja Kunej

Slovenia

Ruprecht Kuner

Germany

Hans Jörg Kunte

Germany

Vasu Kuraparthy

USA

Siavash Kurdistani

USA

Lukasz Kurgan

Canada

Ken Kurokawa

Japan

Tina Kyndt

Belgium

Maris Laan

Estonia

Jean Labarre

France

Joanne Labate

USA

Pierrick Labbé

France

Bernard Labedan

France

Vincent Lacroix

France

Artem Lada

USA

Emmanuel Ladoukakis

Greece

Bernard Lafont

USA

Ulf Lagercrantz

Sweden

Karin Lagesen

Norway

Kaitao Lai

Australia

Dilip Lakshman

USA

Rup Lal

India

Tommy Tsan-Yuk Lam

USA

Fabien Lamaze

Canada

Annee-Marie Lambeir

Belgium

Kathrin Lampert

Germany

Alejandra Landau

Argentina

B. Franz Lang

Canada

David Langenberger

Germany

Gernot Längst

Germany

Cristina Lanzas

USA

Mette Larsen

Denmark

Christer Larsson

Sweden

Justin Graham Lashbrooke

South Africa

Jean-Paul Latge

France

Amparo Latorre

Spain

Sascha Laubinger

Germany

Enrico Lavezzo

Italy

Vladimir Lazarevic

Switzerland

Conxi Lázaro

Spain

Stephane Le Crom

France

Loïck Le Dantec

France

Gaelle Le Goff

France

Karine Gaelle Le Roch

USA

Gael Le Trionnaire

France

Rebecca Leary

USA

Scot Leary

Canada

Michael Leaver

UK

Marc Lebrun

France

Pierre Lechat

France

Erica Leder

Finland

Alygunia Lee

China

Bonghee Lee

Korea South

Charles Lee

USA

Chia Lee

USA

Hane Lee

USA

Hyun Soon Lee

Korea South

Jinwook Lee

USA

Sanghyeob Lee

Korea South

Seon-Woo Lee

Korea South

Suk-Ha Lee

Korea South

Youngsook Lee

Korea South

Tosso Leeb

Switzerland

Carolyn Lee-Parsons

USA

Louis Lefebvre

Canada

Philippe Lefrançois

USA

Stefanie Lehner

Germany

Sigrid Lehnert

Australia

Jianjun Lei

China

Jorge Humberto Leitão

Portugal

Ilia Leitch

UK

Tobias Lenz

USA

Luciana Leomil

Brazil

Olivier Leprince

France

Emmanuelle Lerat

France

Christine Leroux

France

Donald Les

USA

Olivier Lespinet

France

Henry C.M. Leung

Hong Kong

Cathy Levenson

USA

Joshua Levin

USA

Haim Levy

Israel

Randolph Lewis

USA

Semen Leyn

Russian Federation

Natalie Leys

Belgium

Karina Lezirovitz

Brazil

Changchun Li

China

Chaoyang Li

USA

Dalin Li

USA

Dayong Li

China

Fenge Li

China

Genyi Li

Canada

Hongjie Li

China

Hui Li

China

Jingyi Jessica Li

USA

Jinquan Li

China

Jinyun Li

USA

Laigeng Li

China

Lei Li

USA

Maoteng Li

China

Ming Li

USA

Peng Li

USA

Sheng Li

China

Wei Li

USA

Weizhe Li

USA

Wenbin Li

China

Xia Li

China

Xianran Li

USA

Xin Li

USA

Xinguo Li

Australia

Xuan Li

China

Xuewei Li

China

Yongchun Li

China

Zenglu Li

USA

Zhengguo Li

China

Zhigang Li

USA

Chun Liang

USA

Haiying Liang

USA

Wilson Liao

USA

Marc Libault

USA

Francesco Licausi

Italy

David Lightfoot

USA

David Lightfoot

USA

Boon L. Lim

China

Yong Pyo Lim

Korea South

Gipsi Lima Mendez

Belgium

Guangyun Lin

USA

Nianwei Lin

USA

Shuo Lin

USA

Tsan-Piao Lin

Taiwan

Ying Lin

China

Zhenguo Lin

USA

Zhihua Lin

China

Zhongxu Lin

China

Nilton Lincopan

Brazil

Magdalen Lindeberg

USA

King-Hwa Ling

Malaysia

Bryan Linggi

USA

Dirk Linke

Germany

Arimantas Lionikas

UK

Peter Lipke

USA

Damon Lisch

USA

Ryan Lister

Australia

.Xiufan Liu

China

Aizhong Liu

China

Chun-Chi Liu

Taiwan

Don Liu

USA

George Liu

USA

Jian-Feng Liu

China

Jianxiang Liu

China

Jihong Liu

China

Jun-Jun Liu

Canada

Kede Liu

China

Nannan Liu

USA

Pengfei Liu

USA

Pentao Liu

UK

Pingsheng Liu

China

Qingpo Liu

China

Shanlin Liu

China

Shao-Lun Liu

Taiwan

Shengyi Liu

China

Shikai Liu

USA

Suhu Liu

USA

Sunny Liu

USA

Tao Liu

USA

Wusheng Liu

USA

Xiaole Liu

USA

Yanling Liu

China

Zhi-Ping Liu

China

Sabino Liuni

Italy

Inmaculada Llamas

Spain

Danny James Llewellyn

Australia

Manuel Llinas

USA

Christer Löfstedt

Sweden

Maria Logacheva

Russian Federation

Kirk Lohmueller

USA

Nicholas Loman

UK

Sarah London

USA

Arturo Londono Vallejo

France

Manyuan Long

USA

Paul Long

UK

Juan Loor

USA

Camilo Lopez

Colombia

Elena Lopez-Girona

Spain

Rodolfo Lopez-Gomez

Mexico

Luis Vicente Lopez-Llorca

Spain

Belen Lorente-Galdos

Spain

Elgion Loreto

Brazil

Mathias Lorieux

France

Petra Louis

UK

Eliezer Louzada

USA

Ruth Lovering

UK

Hua Lu

USA

Shanfa Lu

China

Alberto Maria Luciano

Italy

Fernandez Lucie

France

Arne Ludwig

Germany

Christian Lueck

Germany

Jingchu Luo

China

Pavlo Lutsik

Germany

Xuebin Lv

China

David Lynn

Ireland

Leslie Lyons

USA

X M

China

Jianxin Ma

USA

Li-Jun Ma

USA

Nyuk Ling Ma

Malaysia

Wujun Ma

Australia

Rodwell Mabaera

USA

Jennifer Macalady

USA

David Macalpine

USA

Stuart Macdonald

USA

Robert L. Mach

Austria

Carlos Machado

USA

Maria De Fatima Machado

Brazil

Masayuki Machida

Japan

David Machugh

Ireland

John Mackay

Canada

Simon Mackenzie

UK

Stephen Mackessy

USA

Michael Macneil

USA

Ross Macphee

USA

Andrew Macrae

Brazil

Frank Madeo

Austria

Ole Madsen

Netherlands

Jeroen Maertzdorf

Germany

Gregory Maes

Belgium

Valeria Mafra

Brazil

Nancy Mah

Germany

Christopher Maher

USA

Tim Mahony

Australia

Robert Maier

USA

Uwe Maier

Germany

Alexis Maizel

Germany

Kira Makarova

USA

Vsevolod Makeev

Russian Federation

Takashi Makino

Japan

Igor Makunin

Russian Federation

Jaleh Malakooti

USA

Andre Malan

France

Jacob Malcom

USA

Rene Malenfant

Canada

Chris Maliepaard

Netherlands

Johan Malmstrom

Sweden

Mickael Malnoy

Italy

Marcos Malosetti

Netherlands

Si Ming Man

USA

Elisabetta Manduchi

USA

Serghei Mangul

USA

Ranjan Mannige

USA

Gerard Manning

USA

Paride Mantecca

Italy

Roberto Mantovani

Italy

Nitin Mantri

Australia

Beatrice Mao

USA

Long Mao

China

Daniel Mapleson

UK

Martial Marbouty

France

Marina Marcet-Houben

Spain

Julian Marchesi

UK

Antonio Marco

UK

Susana N Marcucci Poltri

Argentina

Jim Marden

USA

Laurence Maréchal-Drouard

France

Marc Marenda

Australia

Frank Mari

USA

Tomislav Maricic

Germany

Leonardo Marino-Ramirez

USA

Sanford Markey

USA

Victor Markowitz

USA

Jeffrey Marks

USA

Marilis Marques

Brazil

Tomas Marques-Bonet

Spain

John Martens

Netherlands

Ruth Martin

USA

Sam Martin

UK

Jose Martinez

Spain

Paulino Martínez

UK

Paulino Martínez

Spain

Nilce M Martinez-Rossi

Brazil

David Martinez-Torres

Spain

Isabelle Martin-Verstraete

France

Shinichiro Maruyama

Canada

María Paz Marzolo

Chile

Martin Mascher

Germany

Sergei Maslov

USA

Christopher Mason

USA

Kevin Mason

USA

Andrea Masotti

Italy

Ramon Massana

Spain

Susan Masta

USA

Emma Master

Canada

Anna Maria Mastrangelo

Italy

Kostas Mathiopoulos

Greece

Kazuki Matsubara

Japan

Fumio Matsuda

Japan

Akihro Matsui

Japan

Hideo Matsumura

Japan

Lakshmi Matukumalli

USA

José Tomás Matus

Spain

Mikhail Matz

USA

Felix Mauch

Switzerland

Ujjwal Maulik

India

Julie Maupin-Furlow

USA

Raja Mazumder

USA

David Mazurais

France

John Allan Mccallum

New Zealand

Fiona Mccarthy

USA

Curt Mccartney

Canada

Mark Mcclain

USA

John Mccutcheon

USA

Tara Mcdaneld

USA

Jason Mcdermott

USA

Juan Mcewen

Colombia

Cecilia Mcgregor

USA

Leah Mchale

USA

Michael Mcinerney

USA

Stephanie Mckay

USA

Duane Mckenna

USA

Andrew Mckenzie

UK

Rima Mcleod

USA

Linda Mcloon

USA

Joel Mcmanus

USA

Tim Mcnellis

USA

Steven Mcorist

UK

Rui Medeiros

Portugal

Jp Medema

Netherlands

Marnix Medema

Netherlands

John Meeks

USA

Shawn Mehlenbacher

USA

Angela Mehta

Brazil

Annemarie Meijer

Netherlands

Lee Meisel

Chile

Alexander Mellmann

Germany

Daniel Menendez

USA

Jinling Meng

China

Rajasree Menon

USA

Uwe Menzel

Germany

Enrique Merino

Mexico

Florian Mertes

Germany

Karin J. Metzner

Switzerland

Axel Meyer

Germany

Clifford Meyer

USA

Eli Meyer

USA

Folker Meyer

USA

Jobst Meyer

Germany

Matthias Meyer

Germany

Blake Meyers

USA

Wei Miao

China

Xiangyang Miao

China

Sabrina Micali

Italy

George Michalopoulos

USA

Dominique Michaud

Canada

Johan Michaux

Belgium

Diego Micheletti

Spain

Jan Michiels

Belgium

Tom Michoel

UK

Nicola Miglino

Switzerland

Monika Mihm Carmichael

UK

Denis Milan

France

Massimo Milan

Italy

Dan Milbourne

Ireland

Mattias Mild

Sweden

Alan Mileham

USA

Michel Milinkovitch

Switzerland

Jason Miller

USA

Jeremy Miller

USA

Nicholas Miller

USA

Robert Miller

Brazil

Robert Miller

USA

Janos Minarovits

Hungary

Francisco Juan Miralles

France

Andrey Mironov

Russian Federation

Kazuharu Misawa

Japan

Bibhu Mishra

USA

Manoj Mishra

India

Dorothée Missé

France

Caterina Missero

Italy

Robert Mitchell

USA

Rowan A C Mitchell

UK

Aziz Mithani

Pakistan

T Mito

Japan

Nobutaka Mitsuda

Japan

Balraj Mittal

India

Toutai Mituyama

Japan

Kiyonori Miura

Japan

Toru Miura

Japan

Kenji Mizuguchi

Japan

Kenji Mizuguchi

UK

De Mo

China

Keiichi Mochida

Japan

Keithanne Mockaitis

USA

Thomas Moen

Norway

Cecilia Moens

USA

Perry Moerland

Netherlands

Simone Moertl

Germany

Soren Moestrup

Denmark

James Monaghan

USA

Jorge Mondego

Brazil

Yuki Monden

Japan

Amparo Monfort

Spain

David Monk

Spain

Caroline Montagnani

France

Michael Montague

USA

Clara Montagut

Spain

Claudia Monteiro-Vitorello

Brazil

Claudine Montgelard

France

Jonathan Moore

UK

Robert Moore

Australia

Lucia Morales

France

Aranzazu Moreno

Spain

Richard Morgan

USA

Milana Morgenstern

Spain

Hiroshi Mori

Japan

Naoki Mori

Japan

Peter Morrell

USA

Paul Morris

USA

Janna Morrison

Australia

Cynthia Morton

USA

Mirko Moser

Italy

Sara Mostafavi

USA

Lionel Moulin

France

Isabelle Mouyna

France

Leonie Moyle

USA

Jan Mrazek

USA

Tarek Msadek

France

Wellington Muchero

USA

Gloria Muday

USA

Richard Muench

Germany

Anirban Mukhopadhyay

India

Bertram Müller-Myhsok

Germany

Megan Mulligan

USA

Carolina Munari Rodrigues

Brazil

Monica C Munoz-Torres

USA

Kylie Munyard

Australia

Koji Murai

Japan

Barbara E. Murray

USA

Senthilkumar Murugaiyan

India

Georgi Muskhelishvili

Germany

Peter Myler

USA

Jong-Kuk Na

Korea South

Jose Nacher

Japan

Satoshi Nagai

Japan

Chidananda Nagamangala Kanchiswamy

Italy

Lisa Nagy

USA

Shalima Nair

Australia

Suresh Nair

India

Sushma Naithani

USA

Ichiro Nakagawa

Japan

Ryoko Nakagawa

Switzerland

Kenta Nakai

Japan

Masamichi Nakajima

Japan

Masahiro Nakamura

Japan

Luay Nakhleh

UK

Shigetou Namba

Japan

Indrajit Nanda

Germany

Shawn Narum

USA

June Nasrallah

USA

William Navarre

USA

Alfonso Navas

Spain

Frank Naya

USA

Daniel Neafsey

USA

Aurora Nedelcu

Canada

Alexander J. Nederbragt

Norway

Anna Need

UK

Mark Neff

USA

Enrico Negrisolo

Italy

Antonino Neri

Italy

Chen Siang Ng

Taiwan

Henry Nguyen

USA

Duojiao Ni

USA

Francesco Nicassio

Italy

Krista Nichols

USA

Rick Nicholson

Australia

Scott Nicholson

USA

Kristin Nicodemus

Ireland

Mogens Nicolaisen

Denmark

Marisa F Nicolás

Brazil

Qinghua Nie

China

Einar Nielsen

Denmark

Hans Linde Nielsen

Denmark

Henrik Nielsen

Denmark

Heiner Niemann

Germany

William Nierman

USA

Masahiro Niikura

Canada

Harm Nijveen

Netherlands

Masato Nikaido

Japan

Hans Helmut Niller

Germany

Frank Nilsen

Norway

Tom Ole Nilsen

Norway

Jue Ning

USA

Zemin Ning

UK

Ellen Nisbet

UK

Hiromi Nishida

Japan

Ichiro Nishii

Singapore

Ken Nishikawa

Japan

Takeshi Nishio

Japan

Chikako Nishitani

Japan

Corey Nislow

Canada

Marcelo Nobrega

USA

Hiroaki Noda

Japan

Josselin Noirel

UK

Joseph Norman

Canada

Michael Nothnagel

Germany

Evandro Novaes

USA

Boris Novakovic

Australia

Pavel Novichkov

USA

Katja Nowick

Germany

Norbert Nowotny

Austria

Minou Nowrousian

Germany

Tsukasa Nunome

Japan

Tuula Nyman

Finland

Darren Obbard

UK

Sophie Octavia

Australia

Dong-Ha Oh

USA

Shinsuke Ohba

Japan

John Ohlrogge

USA

Fardod O'Kelly

Ireland

Chinyere Okoro

UK

Carla Oliveira

Portugal

Guilherme Oliveira

Brazil

Mariana Oliveira

Brazil

Brian Oliver

USA

Patrick Ollitrault

France

John Elmerdahl Olsen

Denmark

Michael O'Neill

USA

Suneel Onteru

India

Youko Oono

Japan

Charles Opperman

USA

Laszlo Orban

Singapore

Ludovic Orlando

Denmark

Jamie O'Rourke

USA

Angela Orshinsky

Canada

J. Miguel Ortega

Brazil

Juan Pablo Amelio Ortiz

Argentina

Naoki Osada

Japan

Mizuko Osanai-Futahashi

Japan

Lucy Osborne

Canada

Nir Osherov

Israel

Orla O'Sullivan

Ireland

Yidan Ouyang

China

Ross Overbeek

USA

Tom Owen-Hughes

UK

Maciej Pabijan

Poland

Betty Pace

USA

James Padbury

USA

Abinash Padhi

USA

Gabriel Padilla

Brazil

Amanda Padovan

Australia

Jorge Paiva

Portugal

Suman Pakala

USA

Stela Palii

USA

Oleg Paliy

USA

Subba Palli

USA

Kelli Palmer

USA

Yniv Palti

USA

Tao Pan

USA

Yuchun Pan

China

Franck Panabieres

France

Barry Panaretou

UK

Girdhar Pandey

India

Kristen Panfilio

Germany

Nathan Pankratz

USA

Yvonne Pannekoek

Netherlands

Ralph Panstruga

Germany

Elena Papaleo

Italy

E. Terry Papoutsakis

USA

Emmanuel Paradis

France

Glaucia Paranhos-Baccalà

France

Peter Pare

Canada

Ashwani Pareek

India

Sneh Pareek

India

Hemang Parikh

USA

Christian Parisod

Switzerland

Hee-Bok Park

Korea South

Hyun Park

Korea South

In Kwon Park

USA

Joong-Ki Park

Korea South

Heidi Parker

USA

Julian Parkhill

UK

Isobel Parkin

Canada

Matthew Parks

Australia

Rosa Maria Pascale

Italy

Karla Passalacqua

USA

Marco Passamonti

Italy

Giuseppe Passarino

Italy

Miroslav Patek

Czech Republic

Sonal Patel

Norway

Lavinia Paternoster

UK

Cristian Pattaro

Italy

Josef Patzak

Czech Republic

Steven Paugh

USA

Paul Paukstelis

USA

Catherine Paul

Sweden

Hubert Pausch

Germany

Nathalie Pavy

Canada

Shelley Payne

USA

Cameron Peace

USA

Mary Peacock

USA

Eric Peatman

USA

Thierry Pedrazzini

Switzerland

Mandy Jayne Peffers

UK

Carmen Pelaz

Spain

Pablo Pelegrin

Spain

Mattia Pelizzola

Italy

Julien Pelletier

Sweden

Trevor Pemberton

Canada

Ramona Pena

Spain

Francisco Peñagaricano

USA

Miguel Penalva

Spain

Junmin Peng

USA

You-Liang Peng

China

Michele Perazzolli

Italy

Miguel Perez-Enciso

Spain

Andy Perkins

USA

Antoinette Perry

Ireland

Graziano Pesole

Italy

Stefano Pessina

Italy

Daniel Peterson

USA

Scott Peterson

USA

Daniel Petrovic

Slovenia

Mario Pezzotti

Italy

Vivek Philip

USA

Adam Phillippy

USA

Gregory Phillips

USA

Ruth Phillips

USA

Belen Pico

Spain

Elizabeth Pilon-Smits

USA

Paulo Marcos Pinto

Brazil

Jorge Pinzon

USA

J. Chris Pires

USA

Raul Pirona

Italy

Josep V. Planas

Spain

Matthias Platzer

Germany

Karine Plourde

Canada

Soumita Podder

India

Soumita Podder

India

Lars Podsiadlowski

Germany

Annarita Poli

Italy

Eugenia Poliakov

USA

Adam Polkinghorne

Australia

Igor Ponomarev

USA

Nicolas Pons

France

Siriluck Ponsuksili

Germany

Beatriz Pontes

Spain

Mikhail M. Pooggin

Switzerland

Ilga Porth

Canada

Ezio Portis

Italy

Joël F. Pothier

Switzerland

Deborah Power

Portugal

Helen Poynton

USA

Rolf Prade

USA

Ashwin Prakash

USA

Manoj Prasad

India

Manoj Prasad

India

Randall Prather

USA

Peter Preiser

Singapore

Howard Prentice

USA

Jared Price

USA

Alessandro Prigione

Germany

Alessandro Prigione

Germany

Craig Primmer

Finland

Jaime Prohens

Spain

Andreas Prokesch

Austria

Adrianne Prokupek-Pickett

USA

Mattia Prosperi

USA

Xose Puente

Spain

Marco Punta

UK

Joshua Puzey

USA

Joshua Puzey

USA

Ji Qi

China

Jianjun Qi

China

Qibin Qi

USA

Qian Qian

China

Li-Xuan Qin

USA

Qiwei Qin

China

Deyou Qiu

China

Christian Quack

Germany

Michael Quail

UK

Vera Quecini

Brazil

Joshua Quick

UK

Edwige Quillet

France

Sylvie Marie Anne Quiniou

USA

Fabiola Quintero-Rivera

USA

David Raftos

Australia

Alexander Raikhel

USA

Johannes Rainer

Austria

Gireesh Rajashekara

USA

Om Rajora

Canada

Ravi Rajwanshi

India

Jasna Rakonjac

New Zealand

Ramesh Ramachandran

USA

Srinivasan Ramachandran

Singapore

Yuliaxis Ramayo-Caldas

Spain

Roberto Rangel

USA

Sudha Rao

India

Didier Raoult

France

Marcin Rapacz

Poland

David Rasko

USA

Gurdeep Rastogi

India

Thomas Rattei

Austria

Andrea Rau

France

Laureana Rebordinos

Spain

Umesh Reddy

USA

Venu Reddyvari-Channarayappa

USA

James Reecy

USA

Michael Rehli

Germany

Adam Reid

UK

Valerie Reinke

USA

Adam Reitzel

USA

Jun Ren

China

Gilles Renand

France

Bernhard Renard

Germany

Gabriel Renaud

Germany

Tristan Renault

France

Suzy Renn

USA

Stephen Renshaw

UK

Zhuo Renying

China

Pierre-Yves Rescan

France

Wolfgang Resch

USA

Oleg Reva

South Africa

Tamas Revay

Canada

Fabien Reyal

France

Teresa Reyes

USA

Diego Mauricio Riano-Pachon

Brazil

Jose Ribeiro

USA

Stephen Richards

USA

Vincent Richards

USA

Bryce Richardson

USA

Ruth Richardson

USA

Uwe Richter

Finland

Heathcliffe Riday

USA

Arleen Rifkind

USA

Guillem Rigaill

France

Jouko Rikkinen

Finland

Gonzalo Rincon

USA

Johana Rincones

Brazil

Matthew Rise

Canada

Vikas Rishi

India

Claude Rispe

France

Evelyn Rivera-Toledo

Mexico

Rita Roberti

Italy

Steven Roberts

USA

Gene Robinson

USA

Katie Robinson

Australia

Steve Robinson

Canada

Alexander Robling

USA

Eduardo Rocha

France

Christopher D. Rock

USA

Maria Rosario Rodicio

Spain

Annie Rodolakis

France

Guillermo Rodrigo

Spain

Ricardo Rodriguez De La Vega

Germany

Susana Rodriguez-Navarro

Spain

Francisco Rodriguez-Valera

Spain

Jung-Hye Roe

Korea South

Claire Rogel-Gaillard

France

Robert Roghair

USA

Igor Rogozin

USA

Antonis Rokas

USA

Stephanie Rollmann

USA

Luisa Romao

Portugal

David Romero

Mexico

Junkang Rong

China

Marty Roop

USA

Gail Rosen

USA

Glenn Rosen

USA

Philip Rosenstiel

Germany

Isabelle Rosinski-Chupin

France

Jason Ross

USA

Wilma Ross

USA

David Rossell

Spain

Omar Rota-Stabelli

Italy

Alan Roter

USA

Andrew Roth

Canada

David Roth

USA

Kenneth Rothman

USA

Steven Rothstein

Canada

Klemens Rottner

Germany

Trine Rounge

Norway

Carlos Rovira

Sweden

Kevin Rozwadowski

Canada

David Ruau

UK

Francesca Ruberti

Italy

Carl-Johan Rubin

Sweden

Vicente Rubio

Spain

Matthias Rübner

Germany

Douglas Ruden

USA

Jan Ruijter

Netherlands

Laura Runyen-Janecky

USA

Shivani Ruparel

USA

Paul Rushton

USA

Hege G. Russnes

Norway

Peter Ryan

Australia

Linda Rymarquis

USA

Carla S. Santos

Portugal

Elena Sadchikova

Russian Federation

Avi Sadka

Israel

Richard Saffery

Australia

Geza Safrany

Hungary

Malay Saha

USA

Sudipto Saha

India

Ozgur Sahin

USA

Milton Saier

USA

Atsushi Saito

Japan

Noboru Sakabe

USA

Hiroaki Sakai

Japan

Takashi Sakamoto

Japan

Kazuo Sakka

Japan

Yoshihiro Sakoda

Japan

Poongothai Sakthivel

India

Antonio Salas

Spain

Mohamed Salem

USA

Emili Salo

Spain

Paulo Salomon

Brazil

Silvio Salvi

Italy

Christine Sambles

UK

Michael Sammeth

Spain

Scott Samuels

USA

Diego San Mauro

Spain

Diego San Mauro

UK

Gabino Sanchez Perez

Netherlands

Olivier Sandra

France

Yongming Sang

USA

Vijay Sankaran

USA

Phillip Sanmiguel

USA

Javier Sanzol

Spain

Thomas Sappington

USA

Louis Sarda

France

Daniel Sargent

UK

Daniel Sargent

Italy

Mirella Sari Gorla

Italy

Elena Sarropoulou

Greece

Aaron Sarver

USA

Daimei Sasayama

Japan

Andrea Sass

Belgium

Samuel Sathyanesan Newton

USA

Shusei Sato

Japan

Akira Satoh

Japan

Yutaka Satou

Japan

Barry James Saville

Canada

Samir Sawant

India

Frank Scannapieco

USA

Frank Schacherer

Germany

Joseph Schacherer

France

Katrin Schaefer

Germany

Mary Schaeffer

USA

David Schatz

USA

Michael Schatz

USA

Sven-Eric Schelhorn

Germany

Francesco Paolo Schena

Italy

André Scherag

Germany

Laurent Schibler

France

David Schild

USA

Carl Schildkraut

USA

Enrico Schleiff

Germany

Dorit Schleinitz

Germany

Carl Schmidt

USA

Sheila Schmutz

Canada

Eberhard Schneider

Germany

Tom Schneider

USA

Johannes Schoedel

Germany

Gerald Schoenknecht

USA

Stefan Scholz

Germany

Christian Schönbach

Japan

Irene Schrank

Brazil

Andreas Schreiber

Australia

Hildgund Schrempf

Germany

Declan Schroeder

UK

Patricia Schulte

Canada

Reiner Schulz

UK

Anouk Schurink

Netherlands

Elisabeth Schwartz

Brazil

Russell Schwartz

USA

Wolfgang Schweiger

Austria

Lisa Seeb

USA

Stefan Seefelder

Germany

Ole Seehausen

Switzerland

Ratnam Seelan

USA

Verena Seidl-Seiboth

Austria

David Sela

USA

Lucy Seldin

Brazil

Herve Seligmann

Israel

Francois Seneca

USA

Senapathy Senthilvel

India

Young Rok Seo

Korea South

Bertrand Seraphin

France

Kjell Sergeant

Luxembourg

David Serre

USA

Joao Setubal

USA

Joao Setubal

Brazil

Ning-Yi Shao

USA

Renfu Shao

Australia

Michael Shapero

USA

Cynthia M. Sharma

Germany

Prakash Sharma

India

Tilak Sharma

India

Paul Sharpe

UK

Weifeng She

USA

Andrew Shedlock

USA

Samuel Shelburne

USA

Quan Shen

China

Wen-Hui Shen

France

Xinlian Shen

China

Yao Shen

USA

Yufeng Shen

USA

Samuel Sheppard

UK

Richard Sherva

USA

Peng Shi

China

Tieliu Shi

China

Xiaoli Shi

USA

Xiaoli Shi

USA

Sagiv Shifman

Israel

Shuji Shigenobu

Japan

Chuya Shinzato

Japan

Kenta Shirasawa

Japan

Gautam Shirsekar

USA

Eiichi Shoguchi

Japan

Huixia Shou

China

Qing Yan Shu

China

Arti Shukla

USA

Jeffry Shultz

USA

Izabela Sierocka

Poland

Amaro Silva

Brazil

Herman Silva

Chile

Joana Silva

USA

Roberto Silva

Brazil

Andrew Silver

UK

Francesco Silvestrini

Italy

Sung-Chur Sim

USA

Henner Simianer

Germany

Mark Simmons

USA

Philipp Simon

USA

Ronald Simon

Switzerland

Michel Simonneau

France

Claire Simpson

USA

Luca L. Sineo

Italy

Ambuj Singh

USA

Anil Singh

India

Rama Singh

Canada

Alok Krishna Sinha

India

Diana Six

USA

Nadia Skorupa Parachin

Brazil

Anna Skorupska

Poland

Grethe Skretting

Norway

Jerod Skyberg

USA

Ondrej Slaby

Czech Republic

Steven Slater

USA

Barry Sleckman

USA

Ian Small

Australia

Alison G Smith

UK

Andrew Smith

USA

David Smith

Canada

David Roy Smith

Canada

Jacqueline Smith

UK

Stephen Smith

USA

M Smits

Netherlands

Michaela Smolle

USA

Alan Smulian

USA

Petr Smykal

Czech Republic

Terry Snell

USA

Darren Soanes

UK

Ana Soares

Portugal

Beatriz Sobrino

Spain

Joel Sohlberg

Sweden

Evgeni Sokurenko

USA

Knut Asbjørn Solhaug

Norway

Alexandra Soltész

Hungary

Mehmet Somel

USA

Guisheng Song

USA

Jiuzhou Song

USA

Jun Song

Canada

Qiang Song

USA

Nucharin Songsasen

USA

Tad Sonstegard

USA

María José Soto

Spain

Ana Sousa

Portugal

Ulla Sovio

UK

Ramanathan Sowdhamini

India

Matthew Spangler

USA

Pietro Spanu

UK

Dimitrios Spentzos

USA

Salvatore Spicuglia

France

Fernanda Spies

Brazil

Monika Spiller

Germany

Robert Sprando

USA

Fabio Squina

Brazil

Rohith Srivas

USA

Anuj Srivastava

USA

Kshitij Srivastava

USA

Charley Staats

Brazil

Gary Stacey

USA

Simona Stager

Canada

Boryana Stamova

USA

Dana Stanley

Australia

Spencer Staton

USA

Regine Steegers-Theunissen

Netherlands

Juan Steibel

USA

Katrina Steiling

USA

Nils Stein

Germany

Ursula Steinfort

Chile

Jan Stenlid

Sweden

Burkhard Steuernagel

UK

Marc Stevens

Switzerland

Brian Stevenson

USA

Tim Stinear

Australia

Matthew Stocks

UK

Eric Stone

USA

Jonathan Stone

Australia

Gary Stormo

USA

Gisela Storz

USA

Jeffrey Todd Streelman

USA

Nathaniel Street

Sweden

Gary Strobel

USA

Lisa Stubbs

USA

Frank Stüber

Switzerland

David Studholme

UK

Eva Stukenbrock

Germany

Armin Sturm

UK

Andrew Su

USA

Chunlei Su

USA

Zhen Su

China

Mayte Suarez-Farinas

USA

Senthil Subramanian

USA

Angela Suburo

Argentina

Smita Sudheer

India

Garret Suen

USA

Jung-Pil Suh

Korea South

Deqiang Sun

USA

Xiaowen Sun

China

Yanni Sun

USA

Zhifu Sun

USA

Maximiliano Suster

Norway

John Sved

Australia

Per-Arne Svensson

Sweden

Ronald Swanstrom

USA

Crystal Sweetman

Australia

Luc Swevers

Greece

William Swindell

USA

Marek Switonski

Poland

David Symer

USA

Laszlo Szabados

Hungary

Les Szabo

USA

Karol Szafranski

Germany

Steven Szczepanek

USA

Peter Szovenyi

Switzerland

Zofia Katarzyna Szweykowska-Kulinska

Poland

Moshe Szyf

Canada

Peter-Bram 'T Hoen

Netherlands

Malancha Ta

India

Carlos Taborda

Brazil

John Taggart

UK

Hidetoshi Tahara

Japan

Hideyuki Takahashi

Japan

Tetsumi Takahashi

Japan

Shin Taketa

Japan

Adam Takos

Denmark

Shigeo Takumi

Japan

Manuel Talon

Spain

Javier Tamames

Spain

Koichiro Tamura

Japan

Elly Tanaka

Germany

Mark Tanaka

Australia

Masaru Tanaka

Japan

Ryusei Tanaka

Japan

Toshiko Tanaka

USA

Tsuyoshi Tanaka

Japan

Haibao Tang

USA

Haixu Tang

USA

Jifeng Tang

Netherlands

Patrick Tang

Canada

Shouwei Tang

China

Zhonglin Tang

China

Keiji Tanimoto

Japan

Jose Eduardo Tanus Dos Santos

Brazil

Jun Tao

China

Yong Tao

China

Mika Tarkka

Germany

Deirdre Ellen Kathleen Tarr

USA

Ann Tarrant

USA

Gian Gaetano Tartaglia

Spain

Andreas Tauch

Germany

Graham Teakle

UK

Scott Tebbutt

Canada

Vladimir Teif

Germany

Lucia Teixeira

Brazil

Santuza Teixeira

Brazil

Ross Tellam

Australia

Evelyne Téoulé

France

Nigel Ternan

UK

Javier Terol

Spain

Alexey Terskikh

USA

Andrew Teschendorff

UK

Dawit Tesfaye

Germany

Jens Tetens

Germany

Herve Tettelin

USA

Andreas Teufel

Germany

Georg Thaller

Germany

Louise Thatcher

Australia

Jyothi Thimmapuram

USA

Carmen Thomas

Spain

Clément Thomas

Luxembourg

Jim Thomas

USA

Milt Thomas

USA

Rasmi Thomas

USA

Torsten Thomas

Australia

Andrew Thompson

UK

Anne Thompson

USA

Claudia Elizabeth Thompson

Brazil

Fabiano Thompson

Brazil

Bo Thomsen

Denmark

Snorri Thorgeirsson

USA

Kelsie Thu

Canada

Bala Reddy Thumma

Australia

Bin Tian

USA

Dacheng Tian

China

Qiang Tian

USA

Shufeng Tian

China

Wei Tian

China

Xiangjun Tian

USA

Yingfang Tian

USA

Hagen Tilgner

USA

Angela Ting

USA

Heidi Tissenbaum

USA

Basant Tiwary

India

Raquel Tobes

Spain

David Tobin

USA

Michael Tobler

USA

Douglas Tocher

UK

Katsushi Tokunaga

Japan

Catherine Laure Tomasetto

France

Pietro Tonutti

Italy

Christopher Toomajian

USA

Noel Tordo

France

Ali Torkamani

USA

Jeffrey Townsend

USA

Livio Trainotti

Italy

Nham Tran

Australia

Timothy Tranbarger

France

George Trendelenburg

Germany

Ben Trevaskis

Australia

Martin Trick

UK

Edward Trifonov

Israel

William Trimble

USA

Prateek Tripathi

USA

Matthias Trost

UK

John Trowsdale

UK

Dean Troyer

USA

Vance Trudeau

Canada

Adrian Tsang

Canada

Kenichi Tsuda

Germany

Koji Tsuda

Japan

Kenji Tsuge

Japan

Seiji Tsuge

Japan

Shogo Tsuruta

USA

Zhijian (Jake) Tu

USA

Priscilla Tucker

USA

Christopher Tuggle

USA

Christine Turenne

Canada

Francesca Turroni

Italy

Akhilesh Tyagi

India

Benjamin Tycko

USA

Milton Typas

Greece

Yoshinobu Uemoto

Japan

Durdica Ugarkovic

Croatia

George Uhl

USA

Paul Uhlir

USA

Pekka Uimari

Finland

Cirano José Ulhoa

Brazil

Mauricio Ulloa

USA

Elisabetta Ullu

USA

Gudrun Ulrich-Merzenich

Germany

Gottfried Unden

Germany

Turgay Unver

Turkey

Ralf Uptmoor

Germany

Chris Upton

Canada

Thomas Urban

USA

Claude Urbany

Germany

Turán Péter Ürményi

Brazil

Chisato Ushida

Japan

David Ussery

Denmark

David Ussery

USA

Vladimir Uversky

USA

Giampiero Valè

Italy

Schurdi-Levraud Valerie

France

Tatiana Vallaeys

France

Eric Vallender

USA

Jeffrey Vallet

USA

Claudio Valverde

Argentina

Jose R Valverde

Spain

Harm Van Bakel

Canada

Steven Van Borm

Belgium

Yves Van De Peer

Belgium

Eric Van Der Graaff

Austria

Ida Van Der Klei

Netherlands

Mark Van Der Linden

Germany

Gian Van Der Spuy

South Africa

Allen Van Deynze

USA

Aalt-Jan Van Dijk

Netherlands

Gary Van Domselaar

Canada

Doug Van Hoewyk

USA

Peter Van Loo

UK

Jaap Van Tuyl

Netherlands

Karel H. M. Van Wely

Spain

Carroll Vance

USA

Peter Vandamme

Belgium

Ilse Vandecandelaere

Belgium

Jozef Vanden Broeck

Belgium

C. Vander Schoot

Netherlands

Bingshan Vanderbilt

USA

Jens Vanselow

Germany

Maite Vaslin

Brazil

Roel Veerkamp

Netherlands

Edwin Veldhuizen

Netherlands

Marco Ventura

Italy

Salvador Ventura

Spain

Luca Venturini

Italy

Rosario Vera-Estrella

Mexico

Patricia Veras

Brazil

Ignazio Verde

Italy

Chris Verhofstede

Belgium

Praveen Verma

India

Annaleen Vermeulen

USA

Michiel Vermeulen

Netherlands

Paul Verslues

Taiwan

Kevin J. Verstrepen

Belgium

Ramesh Vetukuri

Sweden

Ana Carolina Vicente

Brazil

Ramon Vidal

Brazil

Alexandre Rezende Vieira

USA

Jorge Vieira

Portugal

Maria Lucia Carneiro Vieira

Brazil

Sara Vieira-Silva

Belgium

Yves Vigouroux

France

Carlos Vilches

Spain

Odile Viltart

France

Raivo Vilu

Estonia

Michel Vincentz

Brazil

Patrick Viollier

Switzerland

Agustin Vioque

Spain

Jugsharan Virdi

India

Marleen Visker

Netherlands

Rui Vitorino

Portugal

Nicola Vitulo

Italy

Melane Alethea Vivier

South Africa

Peter Vlasov

Spain

Lila Vodkin

USA

Birgit Voigt

Germany

Christian Voigt

Germany

Adrian Vojnov

Argentina

Diter Von Wettstein

USA

Igor Vorechovsky

UK

Frank-Jorg Vorholter

Germany

Claire Vourc'H

France

Agnieszka Waclawik

Poland

Simon Waddell

UK

Simon Wagstaff

UK

Virginia Walbot

USA

Geoff Waldbieser

USA

Levi Waldron

USA

Alan Walker

UK

Brian Walker

UK

John Walker

USA

Dennis Wall

USA

Emma Wall

USA

Jason Wallace

USA

Rob Wallace

USA

Alison Waller

Germany

Andrew Waller

UK

Peter G. Walley

UK

Juline Walter

Brazil

Lutz Walter

Germany

James Walters

USA

Chenghui Wang

China

Chengshu Wang

China

Chunlai Wang

China

Claire Qiufan Wang

USA

Daowen Wang

China

Guang-Zhong Wang

USA

Hurng-Yi Wang

Taiwan

Jianmin Wang

China

Jianping Wang

USA

Junwen Wang

China

Liangjiang Wang

USA

Lijun Wang

China

Lily Wang

USA

Li-Shun Wang

China

Mingyi Wang

USA

Ning Wang

USA

Shaolin Wang

USA

Wen Wang

China

Xiangfeng Wang

USA

Xiaojie Wang

China

Xiao-Wei Wang

China

Xiaowu Wang

China

Xiping Wang

China

Xiyin Wang

USA

Ying Wang

USA

Ying Wang

China

Yuanchao Wang

China

Zhiquan Wang

Canada

Zhong Wang

USA

Dierk Wanke

Germany

Kevin Wanner

USA

Aoife Ward

Germany

Judson Ward

USA

Hans-Jörg Warnatz

Germany

James Wasmuth

Canada

Trudy Wassenaar

Germany

Shugo Watabe

Japan

Elizabeth Waters

USA

Chris Watkins

UK

Mick Watson

UK

Hongbo Wei

China

Ming Ming Wei

China

Yangdou Wei

Canada

Thomas Weide

Germany

Steffen Weigend

Germany

Zasha Weinberg

USA

Virginia Weis

USA

Joachim Weischenfeldt

Germany

Lauren Weiss

USA

Roy Welch

USA

Rachel Wells

UK

Li Wen-Xue

China

Elizabeth Weretilnyk

Canada

Andreas Werner

UK

Thomas Werner

Germany

Johan Westerhuis

Netherlands

Hannah Wexler

USA

Tom Wheeler

New Zealand

Matthew White

USA

Emma Whitelaw

Australia

Ryan Whitford

Australia

Ralph Wiedmann

USA

Stefan Wiemann

Germany

Pam Wiener

UK

Christoph Wierling

Germany

Sharon Wildes

USA

Brian Wilhelm

Canada

Brian Wilhelm

UK

Matthew Wilkerson

USA

Olivia Wilkins

USA

Jamie Wilkinson

UK

Rob Willems

Netherlands

Christie Williams

USA

Anna-Lise Williamson

South Africa

R Alan Wilson

UK

Richard Wilson

USA

Milo Wiltbank

USA

Ernst A. Wimmer

Germany

Klaus Wimmers

Germany

Patrick Wincker

France

Astrid Wingler

UK

Michael Wink

Germany

Sylke Winkler

Germany

Craig Winstanley

UK

Christine Winter

Germany

Aleksandra Wisłowska -Stanek

Poland

Dieter Wolf

USA

Yuri Wolf

USA

Emily Wong

Australia

Andrew Wood

USA

David Wood

USA

Stephen Wright

Canada

Wasco Wruck

Germany

Harry Wu

Sweden

Shu-Hsing Wu

Taiwan

Wenting Wu

USA

Martin Wubben

USA

Hong-Fei Xia

China

Qingyou Xia

China

Xianchun Xia

China

Xinli Xia

China

Wenzhong Xiao

USA

Xinshu Xiao

USA

Dan Xie

USA

Jun-Feng Xie

China

Zhixin Xie

USA

Wang Xin

China

Yi Xin

China

Yongzhong Xing

China

Anlong Xu

China

Dong Xu

USA

Fuyu Xu

USA

Steven Xu

USA

Tianjun Xu

China

Yi Xu

China

Hongyu Xue

USA

Zhao Xue

China

Ramon Xulvi-Brunet

USA

Gitanjali Yadav

India

Jaya Parkash Yadav

India

Masumi Yamagishi

Japan

Hirotaka Yamaguchi

Japan

Riu Yamashita

Japan

Raghunatha Yammani

USA

Guijun Yan

Australia

Koon-Kiu Yan

USA

Wei Yan

USA

Fan Yang

USA

Jean Yang

Australia

Jilong Yang

China

Shihui Yang

USA

Weidong Yang

China

Xiao Yang

USA

Xiaohong Yang

China

Yaodong Yang

China

Yunfeng Yang

China

Zujun Yang

China

Henry Yangh

Singapore

Jia-Long Yao

New Zealand

Quanhong Yao

China

Yuji Yasukochi

Japan

Xingguo Ye

China

Yang Yen

USA

Craig Yendrek

USA

Gwan-Su Yi

Korea South

Qiu Yi

China

Changjun Yin

China

Han Yingpeng

China

Han Yingpeng

China

Jeffrey Yoder

USA

Kentaro Yoshida

UK

Tim Yoshino

USA

Philip Young

South Africa

Rob Young

UK

Bin Yu

USA

Jingjuan Yu

China

Jiujiang Yu

USA

Jun Yu

China

Oliver Yu

USA

Qingyi Yu

USA

Jiazheng Yuan

USA

Lixing Yuan

China

Gen Hua Yue

Singapore

Junming Yue

USA

Ming Yue

USA

Manuel Yúfera

Spain

Chiou-Hwa Yuh

Taiwan

Zhong Yun

USA

Pui Yi Yung

Singapore

Arnaldo Zaha

Brazil

Federico Zambelli

Italy

Dani Zamir

Israel

Rafael Zardoya

Spain

Ignacio Zarra

Spain

Egija Zaura

Netherlands

Mihaela Zavolan

Switzerland

Dmitri Zaykin

USA

Alex Zelikovsky

USA

Igor Zelko

USA

Tomasz Zemojtel

Germany

Karsten Zengler

USA

Philipp Zerbe

Canada

Daniel Zerbino

USA

Jixian Zhai

USA

Aibin Zhan

China

Xiangjiang Zhan

UK

Yiping Zhan

USA

Aimin Zhang

China

Baohong Zhang

USA

Chuan-Xi Zhang

China

Clarence K. Zhang

USA

Deshui Zhang

USA

Guofan Zhang

China

Hong Zhang

USA

Hongbin Zhang

USA

Hui Zhang

China

Jinfa Zhang

USA

Kun Zhang

USA

Li Zhang

USA

Lixin Zhang

China

Meiping Zhang

USA

Rui Zhang

USA

Rui Zhang

China

Shanshan Zhang

USA

Shuqun Zhang

USA

Xianlong Zhang

China

Xiquan Zhang

China

Xiuren Zhang

USA

Xiurong Zhang

China

Xuegong Zhang

China

Xuehong Zhang

China

Yan Zhang

China

Yang Zhang

USA

Yanming Zhang

USA

Yong Zhang

China

Yu-Zhong Zhang

China

Zhengsheng Zhang

China

Zhongyang Zhang

USA

Ziding Zhang

China

Jianguo Zhao

China

Min Zhao

USA

Qiao-Ling Zhao

China

Quanzhi Zhao

China

Shancen Zhao

China

Shu-Hong Zhao

China

Yi Zhao

China

Zhongming Zhao

USA

Chengchao Zheng

China

Shijun Zheng

China

Wenguang Zheng

USA

Wenguang Zheng

USA

Cong-Zhao Zhou

China

Gongke Zhou

China

Huaijun Zhou

USA

Lili Zhou

China

Man Zhou

USA

Qing Zhou

USA

Weijun Zhou

China

Wengang Zhou

USA

Xin Zhou

China

Yaoqi Zhou

USA

Zunchun Zhou

China

Baoli Zhu

China

Guan Zhu

USA

Jianhua Zhu

USA

Qian-Hao Zhu

Australia

Yitan Zhu

USA

Igor Zhulin

USA

Stefan Zimmermann

Germany

Naomi Ziv

USA

Didier Zoccola

Monaco

Gabriele Zoppoli

Italy

Sergey Zotchev

Norway

Andrea Zuccolo

Italy

Jurica Zucko

Croatia

